# CRMP5 Antibodies—Diagnostic Challenges

**DOI:** 10.3389/fneur.2021.729075

**Published:** 2021-09-22

**Authors:** Cecilie Totland, Mette Haugen, Christian Vedeler

**Affiliations:** ^1^Department of Neurology, Haukeland University Hospital, Bergen, Norway; ^2^Neuro-SysMed, Department of Neurology, Haukeland University Hospital, Bergen, Norway; ^3^Department of Clinical Medicine, University of Bergen, Bergen, Norway

**Keywords:** paraneoplastic neurological disease, CRMP5 antibody, peripheral neuropathy, cerebellar ataxia, lung cancer

## Abstract

CRMP5-associated paraneoplastic neurological syndromes (PNS) are rare, and only few studies describe larger cohorts of patients with CRMP5 antibodies. We have included 24 patients with CRMP5 antibodies and compared clinical findings with diagnostic findings from two different line assays (Ravo and Euroimmun), staining of cerebellar sections and results of a newly developed cell-based assay for detection of CRMP5 antibodies, CRMP5-CBA. We found that peripheral neuropathy and cerebellar ataxia together with lung cancer were the most common diagnoses associated with CRMP5 antibodies. CRMP5-CBA was easy to perform, identified all relevant cases for CRMP5-associated PNS and is therefore a valuable add-on for verification of CRMP5 positivity in diagnosis of PNS.

## Introduction

CRMP5 antibodies were first described by Honnorat et al. who called them anti-CV2 (1). These antibodies were found in sera from some patients with paraneoplastic neurological syndromes (PNS) and stained the cytoplasm and processes of oligodendrocytes in the brain stem, spinal cord and cerebellar white matter (1). The antigen was later identified as collapsin response mediator protein 5 (CRMP5), a protein involved in neurite development (2).

PNS commonly associated with CRMP5 antibodies include Lambert-Eaton myasthenic syndrome, limbic encephalitis, encephalomyelitis, cerebellar ataxic syndrome and peripheral neuropathy (1, 3, 4). An underlying cancer can be identified in about 73% of patients with CRMP5 antibody associated PNS (5), and CRMP5 antibodies often coexist with other paraneoplastic antibodies, most commonly anti-Hu (3, 4).

Lung cancer, especially small cell lung cancer (SCLC), and thymoma are the most frequent malignancies found in patients with CRMP5 antibodies (3, 4, 6, 7). CRMP5 is universally expressed in SCLC (6) and CRMP5 antibodies have also been identified in ~5% of the patients with SCLC without PNS (8). Further, 12% of all patients with thymoma and myasthenia gravis have CRMP5 antibodies (8), even though CRMP5 expression has not been found in thymus or thymoma either in patients with CRMP5 antibodies or those without (5).

Immunohistochemical staining with patient sera on fixed rat cerebellar tissue or commercial line assays are the preferred techniques for detection of CRMP5 antibodies. A positive finding in one test should be confirmed by another test and compared with clinical findings before a diagnosis is set. That there are currently only two valid ways to detect CRMP5 antibodies represents several problems. Firstly, CRMP5 antibodies are best detected on rat cerebellar tissue from rats transcardiacally perfused with paraformaldehyde (PFA), and further post fixation of cerebellum in PFA (1). This technique can be challenging to perform at many diagnostics laboratories, as not all have proper animal facilities for such methods. Secondly, commercial available line assays are easier to perform, but recent studies have highlighted that these assays often pick up to many false positives. For CRMP5 it has been estimated that about 50% of all positive findings are false positive (9, 10), so an easy to perform validation assay is much needed.

## Methods

### Patient Selection

In the period 2003–2021, 35,553 patient sera and cerebrospinal fluid (CSF) samples were analyzed for paraneoplastic antibodies at the Neurological Research Laboratory, Haukeland University Hospital, Bergen, Norway. Of these, 36 sera/CSF (24 patients) were positive for CRMP5 antibodies on the 14 PNS line assay from Ravo Diagnostika and were included in this study. These samples were further analyzed using EUROLINE PNS 12 Ag, by indirect immunofluorescence on rat cerebellar sections, and by a newly developed cell-based assay for detection of CRMP5 antibodies (CRMP5-CBA) produced by Euroimmun. Clinical data were obtained from referring neurologists. The study was approved by the regional ethics committee (#242339) as a quality assessment study.

### Line Assay

Two commercially available line assays were used for initial screening for onconeural antibodies. The PNS 14 Line Assay (Ravo Diagnostika, #PNS14-003) includes 14 different antigens for PNS: GAD65, HuD, Yo, Ri, CV2/CRMP5, amphiphysin, Ma1, Ma2, SOX1, Tr/DNER, Zic4, titin, recoverin and Protein Kinase C γ. The EUROLINE PNS 12 Ag (Euroimmun, #DL1111-1601-7-G) includes 12 different antigens for PNS: amphiphysin, CV2/CRMP5, Ma2, Ri, Yo, Hu, recoverin, SOX1, titin, Zic4, GAD65 and Tr/DNER. Serum and CSF samples from 24 patients were analyzed in both line assays following the manufacturer's instructions. Two independent investigators graded band intensities from + (weakly positive) to + + + (strongly positive), compared to a positive control sample (+ + +).

### Indirect Immunofluorescence on Rat Cerebellar Sections

Wistar Hannover GLAST rats were anesthetized and transcardiacally perfused with ice-cold 4% paraformaldehyde (PFA) in PBS. Brains were post-fixed (24 h, 4°C) in PFA, then incubated with 18% sucrose in PBS (72 h, 4°C), snap-frozen, and cut into 10-μm parasagittal sections on a cryostat. Heat-induced antigen retrieval was performed in a 2100 Antigen retriever in Diva Decloaker buffer solution (Biocare Medical, #DV2004MX). Sections were blocked in 0,2% bovine serum albumin (BSA) and 1% Triton X-100 in PBS (2 h, room temperature) and incubated over night at 4 °C with patient samples diluted 1:500 and rabbit-anti-CRMP5 (1:200, Abcam, #AB36203) in blocking solution. The sections were then washed with PBS and incubated with secondary antibody (Alexa Fluor^®^ 488 goat anti-human IgG, Thermo Fisher Scientific, #A-11013, and Alexa Fluor^®^ 594 goat anti-rabbit, Thermo Fisher Scientific, #A11012) diluted 1:100 in blocking buffer for 90 min at room temperature. Sections were then washed in PBS and mounted with Fluoromount-G (Thermo Fisher Scientific, #00-4958-02) and examined by immunofluorescence. Two independent investigators evaluated the results. All procedures were performed according to the National Institutes of Health Guidelines for the Care and Use of Laboratory Animals Norway (FOTS 20135149/20157494/20170001).

### CRMP5 Cell-Based Assay

Anti-CV2/CRMP5 IIFT (#FA 1119-1010-51, Euroimmun) is a test kit from Euroimmun that is not commercially available yet. It is a cell-based assay with HEK293 cells transfected with CRMP5. The kit consist of slides, and each slide contains 10 biochips. Each chip has one field with transfected cells and one field with untransfected cells. The kit was used according to the manufacturer's instructions. Briefly, serum samples were diluted 1:10 and 1:100 in sample buffer. When only CSF was available, this was tested undiluted 1:1. Sample (30 μl) was added to each biochip and incubated at room temperature for 30 min. To verify that serum stained only the CRMP5-positive cells, a co-staining with the rabbit anti-CRMP5 antibody was performed. Slides were then washed with phosphate-buffered saline containing 0.2% Tween 20 (PBS-Tween 20) for 5 min at room temperature, before incubation with secondary antibody (Alexa Fluor 488 goat anti-human IgG and Alexa Fluor 594 goat-anti rabbit (1:500, 30 min, RT). Slides were rinsed with PBS-Tween 20, and mounted on a glass coverslip. The cut-off for anti-CV2/anti-CRMP5 was set to 1:100, as advised by the manufacturer. Sera from 25 CRMP5 negative patients were included as negative controls. Two independent investigators evaluated the results.

## Results

Clinical and diagnostic findings of the 24 patients (17 females and 7 males, mean age 67 years) are presented in Table 1. Lung cancer was the cancer most frequently associated with CRMP5 antibodies (14 patients, 58%) and peripheral neuropathy (sensory-motor neuropathy) was the most prevalent neurological diagnosis (10 patients, 42%) associated with such cancer. Cerebellar ataxia was diagnosed in three patients with lung cancer and one patient with lymphoma. One patient had thymoma and myasthenia gravis, and one patient had neuroendocrine carcinoma and encephalitis. No tumor was found in seven patients (30%) that tested positive for CRMP5 antibodies by Ravo line assay. These patients showed a broader spectrum of neurological diseases, including peripheral neuropathy, cerebellar ataxia, cranial neuropathy, brain infarction, myalgia and radiological isolated syndrome.

**Table 1:** Diagnostic and clinical findings in 24 patients with CRMP5 antibodies.

**Nr**	**Sex, age**	**Line assay RAVO**		**Lineassay Euroimmun**		**Cerebellar sections**	**CBA**	**Neurological symptoms**	**Cancer**
		***CRMP5***	***Other AB***	***CRMP5***	***Other AB***				
1	M, 68	+ + +		+ + +		+	1/100	Peripheral neuropathy	Lung cancer
2	F, 62	+		++		+	1/10,000	Peripheral neuropathy	Lung cancer
3	F, 75	++	Hu/Sox1	++	Hu/Sox1	+	1/10,000	Peripheral neuropathy	Lung cancer
4	F, 71	++	Sox1	-	Amph	-	1/10,000	Peripheral neuropathy	Lung cancer
5	F, 68	++	Hu	++	Hu/Zic4	+	1/100,000	Peripheral neuropathy	Lung cancer
6	F, 78	+ + +	Amph	+ + +	Amph	+	1/100,000	Peripheral neuropathy	Lung cancer
7	F, 74	+ + +		+ + +		+	1/100,000	Peripheral neuropathy	Lung cancer
8	M, 68	++		+		-	1/100,000	Peripheral neuropathy	Lung cancer
9	F, 61	++	Sox1	++	Sox1	+	1/10,000	Peripheral neuropathy	Lung cancer
10^*^	F, 78	+ + +		++	Recoverin	-	1/10,000	Peripheral neuropathy	Lung cancer
11	F, 70	++	Hu	+	Hu	-	1/100,000	Optic neuritis	Lung cancer
12^*^	F, 76	+ + +		++		+	1/1,000	Cerebellar ataxia	Lung cancer
13	M, 70	+ + +	Amph	++	Amph	+	1/10,000	Cerebellar ataxia	Lung cancer
14	F, 69	+ + +	Hu	+ + +	Hu	+	1/100,000	Cerebellar ataxia	Lung cancer
15^*^	F, 68	++		+		+	1/10,000	Cerebellar ataxia	Lymphoma
16	M, 86	+	Ri	+	Sox1/Ri	-	1/100,000	Encephalitis	Neuroendocrine carcinoma
17	F, 60	++		+ + +		+	1/1,000	Myastenia gravis	Thymoma
18	F, 84	++		++		+	1/100,000	Cerebellar ataxia	No
19	M, 66	+ + +	Hu/Sox1	+ + +	Hu/Recoverin	+	1/10,000	Peripheral neuropathy	No
20	F, 43	+		-		-	1/100	Radiological isolated syndrome	No
21^*^	M, 71	++		-		-	1/10	Brain infarction	No
22	K, 23	++		++		-	-	Brain infarction	No
23	F, 69	+		+		-	-	Cranial neuropathy	No
24	M, 61	+		-		-	-	Myalgia	No

* *, CSF.*

*+ - + + +, intensity score for line immunoassays; +, weakly positive; ++, medium positive; + + +, strongly positive. Immunostaining of cerebellar sections is markerd as + (positive) or – (negative). Titers are indicated for the cell-based assay (CBA), while negative staining is indicated with -. Amph, Amphiphysin. Peripheral neuropathy = sensorimotor polyneuropathy. Follow-up of the cancer negative patients was > 2 years except for patient #21 and #22 with 1 year follow-up*.

In 14 patients (58%) CRMP5 antibodies were the only antibodies identified by the Ravo line assay, while in the other 10 patients (42%) additional antibodies were found. The most common antibodies co-expressed with CRMP5 according to the Ravo line assay were Hu (5 patients, 21%), SOX1 (4 patients, 17%), amphiphysin (2 patient, 8%) and Ri (1 patient, 4%). In two of the patients (8%), both Hu and Sox1 were co-expressed with CRMP5. There were some small differences in the prevalence of additional antibodies identified by Ravo and Euroimmun line assays (Table 1). Cancer was present in all but one of the patients with multiple antibodies, while cancer was detected in only seven of the 13 patients (54%) with only CRMP5 antibodies. Peripheral neuropathy (4 patients) and cerebellar ataxia (3 patients) were the most common PNS seen in patients with only CRMP5 antibodies and the patient with myasthenia gravis and thymoma was only positive for CRMP5.

The level of intensity of the line assays was evaluated by two independent researchers and varied from weak (+) to high intensity (+ + +). Five patients were graded as low intensity, 11 patients as medium intensity and eight patients were rated as high intensity by Ravo line assay. Four of these samples were negative by Euroimmun line assay, two of which were scored as low intensity by Ravo line assay, and two as medium intensity. Apart from these, the correlation in intensities between the two line assays was good (Table 1).

**Figure 1:**
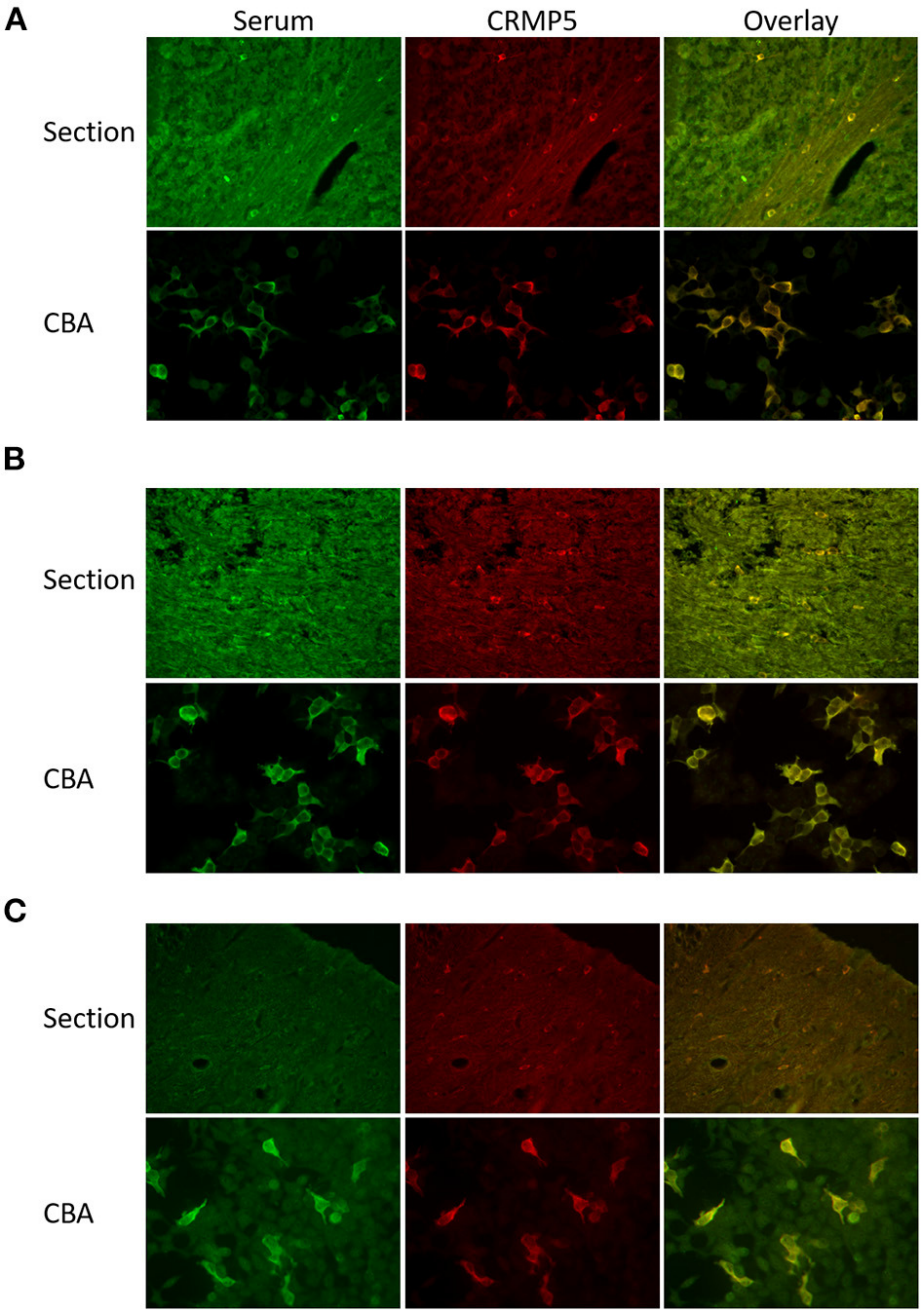
Comparison of CRMP5 staining patterns in cerebellar sections and CRMP5-CBA. **(A)** Section: Serum from a patient with thymoma and myasthenia gravis stains oligodendrocytes in cerebellar white matter (green). A rabbit anti-CRMP5 antibody (red) stains the same cells. Overlay seen in yellow. CBA: Patient serum (green) specifically detect CRMP5-transfekted HEK293 cells. Anti-CRMP5 antibody (red) is used to detect CRMP5 positive cells. Overlay seen in yellow. **(B)** Section: Serum from a patient with peripheral neuropathy, lung cancer, Hu, Zic4, and CRMP5 antibodies. The CRMP5 signal is masked in the additional staining of the other antibodies in the patient serum (green). Co-staining with rabbit anti-CRMP5 antibody (red). The overlay shows that the serum also detects CRMP5 positive oligodendrocytes. CBA: The serum (green) stains CRMP5 positive cells in the CRMP5-CBA. Rabbit anti-CRMP5 (red) is used to verify CRMP5 positive cells. Overlay seen in yellow. **(C)** Serum from a patient with lung cancer and peripheral neuropathy (green) is negative in cerebellar sections.

Detection of CRMP5-positive oligodendrocytes in cerebellar sections can be difficult to identify. If patients sera contain multiple antibodies, the CRMP5 staining can also easily be masked by other antibody staining. To increase the specificity of the oligodendrocyte staining, we used a commercial rabbit anti-CRMP5 antibody for co-staining (Figure 1). No CRMP5 staining was observed in 10 of the patient sera, while positive staining was observed in 14 patients (Table 1).

Twenty-one of the 24 patient sera were positive by CRMP5-CBA with a titer ranging from 1/10 to 1/100,000. None of the sera/CSF stained untransfected HEK293 cells, and no background staining was found in the 25 CRMP5 negative sera. No tumor was detected in the patients with a titer below 1/100. Seven of the sera that were positive by CRMP5-CBA were not positive in cerebellar sections, and all but one of these had an associated cancer. Figure 1 shows a comparison of CRMP5 staining patterns in cerebellar sections and CRMP5-CBA. CRMP5-CBA identified CRMP5 antibodies in a patient that also showed clear CRMP5 reactivity in cerebellar sections (Figure 1A), in a patient with multiple antibodies where the CRMP5 signal was masked by additional staining of other antibodies (Figure 1B), and in a patient that were negative on cerebellar sections, but clearly positive by CRMP5-CBA (Figure 1C).

## Discussion

We evaluated commonly used assays for detection of CRMP5 antibodies in sera and CSF from 24 patients that were identified as positive for CRMP5 antibodies by the Ravo line assay. We compared these results with those obtained using the Euroimmune line assay, immunofluorescence of cerebellar sections and a newly developed cell based assay for detection of CRMP5 antibodies (CRMP5-CBA), as well as with clinical data. There are also other tests commonly used for detecting paraneoplastic antibodies, like assays from Athena Diagnostics or assays used by the Mayo Clinic Laboratories, but there are to our knowledge no studies comparing such assays with the assays from Ravo or Euroimmun.

An associated tumor was found in 71% of the patients, with lung cancer being most prevalent. This in accordance with previous studies that have found that lung cancer and thymoma are most often associated with CRMP5 antibodies (3, 5, 7, 8). Further, we found that peripheral neuropathy was the most often reported PNS associated with anti-CRMP5 followed by cerebellar ataxia, which is also in accordance with previous reports (4, 6). We found additional paraneoplastic antibodies in 10 of the sera and showed that Hu and Sox1 antibodies were those most often identified. In two cases, both Hu and SOX1 antibodies appeared together with CRMP5, which is also in line with previous studies (3, 11). Identification of other autoantibodies co-expressed with CRMP5 is important as peripheral neuropathy has also been associated with Hu and Sox1 antibodies (12–14) and encephalopathy with Ri antibodies (15).

The Euroimmun line assay did not detect four of the 24 positive sera identified by the Ravo line assay, otherwise there was a good correlation between the two assays. This discrepancy might be explained by differences in how the recombinant proteins are produced. While Ravo uses a Baculovirus expression system for expressing full length CRMP5, Euroimmun expresses their proteins in a bacterial expression system.

Most laboratories use commercial line assays and /or immunohistochemistry to detect CRMP5 antibodies. While line assays are easy to perform, they can give false positive results (9, 10, 16). Therefore, another confirmatory test is needed for the line assays. Immunohistochemistry on cerebellar/brain stem tissue has so far been the preferred verification method for anti-CRMP5. However, this technique requires that the tissue is fixed in a specific, time-consuming way (1) which makes it not readily available in many laboratories. Further, even when the tissue is correctly fixed, identification of the CRMP5 positive oligodendrocytes can be difficult, as we show in our study. CRMP5 is expressed in a subpopulation of oligodendrocytes that are scarce in the white matter and in the brain stem. Hence, positive staining can easily be missed, especially if this staining is masked by staining of additional antibodies. In our study, we could detect positive staining in cerebellar sections only in 14 of the 24 sera/CSF, which suggests a significant under-diagnosis of CRMP5 positivity.

In view of the laborious nature of immunohistochemically analysis, we have substituted this technique with a newly developed assay, CRMP5-CBA. The CRMP5-CBA was positive for 21 of the 24 sera that were CRMP5 positive by the Ravo line assay. One of the 21 patient had a titer of 1/10 in the CRMP5-CBA. This is below serum cut-off defined by Euroimmun. However, we only had CSF from this patient and therefore interpreted the result as positive, as it is likely that the CRMP5 antibody level would be higher in a corresponding serum. Since the clinical diagnosis of this patient is brain infarct, it can be assumed that this is a false positive test even though loss of CRMP5 has been associated with brain ischemia in mice (17).

The rate of antibody detection has been shown to increase when both serum and CSF is tested (18). We only had complementing serum and CSF samples for four patients. In all cases, both serum and CSF were positive for CRMP5 antibodies (data not shown). Since the CRMP5-CBA has been optimized for serum testing, we chose to use the serum results in the cases where both serum and CSF were available.

No tumor was detected in seven of the 24 patients. Five of these had a follow up of more than 2 years, while two of the patients had a follow-up of 1 year. We cannot rule out that these two patients could develop cancer at a later time point. All cancer negative patients were CRMP5 antibody positive by the Ravo line assay and four were positive by the Euroimmun line assay whereas three were negative by CRMP5-CBA. Two of these cancer negative patients were strongly positive by CRMP5-CBA and IHC, and one patient had corresponding Hu antibodies by the two line assays. However, no cancer was found by PET scan in this patient (follow up of more than 10 years). Therefore, CRMP5 antibodies are not always correlated with a detectable tumor. Both patients had peripheral neuropathy or cerebellar ataxia, which are most often associated with anti-CRMP5. The other cancer negative patients had diagnoses that are not commonly associated with these antibodies (4). We do not have data on the rate of false-positives for CRMP5-CBA and IHC, but our data shows a good correlation between positive findings and clinical symptoms. Our CRMP5-negative control samples did not stain the CRMP5-CBA. To verify the rate of false-positives a larger control material is needed.

An in-house CRMP5-CBA assay has also been reported previously (19). Using this assay, the authors found that that four of 53 (7.5%) sera being positive by immune-histochemistry and negative by commercial line assays, were positive using their in-house CRMP5-CBA. Whether CRMP5-CBA is more sensitive than the commercial line assays is yet unclear. In our hands, screening sera/CSF for paraneoplastic antibodies by commercial line assays still requires confirmation by another immune assay. For CRMP5 detection, we found that the commercial CBA was more sensitive than immunohistochemistry and we therefore consider it a valuable add-on for verification of CRMP5 positivity in diagnosis of PNS.

## Data Availability Statement

The original contributions presented in the study are included in the article/supplementary material, further inquiries can be directed to the corresponding author/s.

## Ethics Statement

The studies involving human participants were reviewed and approved by Regional ethics committee, Bergen, Norway (#242339). Written informed consent for participation was not required for this study in accordance with the national legislation and the institutional requirements.

## Author Contributions

CT was involved in design of the study, evaluated results, and wrote the manuscript. MH was involved in the design of the study, collected all the data, evaluated the results, and revised the manuscript. CV was involved in the design of the study, evaluated results, and was involved in writing the manuscript. All authors have approved the final version of the manuscript.

## Funding

This study was funded by Helse Vest, project # F-12187.

## Conflict of Interest

The authors declare that the research was conducted in the absence of any commercial or financial relationships that could be construed as a potential conflict of interest.

## Publisher's Note

All claims expressed in this article are solely those of the authors and do not necessarily represent those of their affiliated organizations, or those of the publisher, the editors and the reviewers. Any product that may be evaluated in this article, or claim that may be made by its manufacturer, is not guaranteed or endorsed by the publisher.
